# Entanglement dynamics in the presence of controlled unital noise

**DOI:** 10.1038/srep10796

**Published:** 2015-06-10

**Authors:** A. Shaham, A. Halevy, L. Dovrat, E. Megidish, H. S. Eisenberg

**Affiliations:** 1 Racah Institute of Physics, Hebrew University of Jerusalem, Jerusalem 91904, Israel

## Abstract

Quantum entanglement is notorious for being a very fragile resource. Significant efforts have been put into the study of entanglement degradation in the presence of a realistic noisy environment. Here, we present a theoretical and an experimental study of the decoherence properties of entangled pairs of qubits. The entanglement dynamics of maximally entangled qubit pairs is shown to be related in a simple way to the noise representation in the Bloch sphere picture. We derive the entanglement level in the case when both qubits of a Bell state are transmitted through any arbitrary unital Pauli channel, and compare it to the case when the channel is applied only to one of the qubits. The dynamics of both cases was verified experimentally using an all-optical setup. We further investigated the evolution of partially entangled initial states. Different dynamics was observed for initial mixed and pure states of the same entanglement level.

Quantum entanglement is an essential ingredient in many quantum information tasks. It is vital for the implementation of quantum protocols such as quantum teleportation and dense coding, as well as for other computational schemes^1^. Decoherence — the undesired coupling of a quantum system to other non-accessible systems, results in quantum noise which reduces the degree of entanglement that the system of interest possesses. This in turn can hinder the success of quantum information protocols. Therefore, the characterization of entanglement dynamics under the influence of decohering processes is required for any future realization of these quantum information applications.

There are two main approaches for studying the dynamics of entanglement. In the first, a specific model which is a result of a specific physical implementation of noise is considered, and the time evolution of the system is studied^2^,^3^,^4^,^5^. In the second approach, dynamics is studied more generally: entanglement is calculated from the knowledge of the noise parameters and the initial state, disregarding its time evolution^6^,^7^,^8^,^9^. Some previous works have focused on the specific moment where entanglement disappears, referring to it as the sudden death point if entanglement vanishes while local coherence still prevails^10^,^11^.

Usually, when the effects of a decohering channel on an entangled system are studied, two questions are raised. The first is the general binary question: does the given noisy channel preserves or breaks the entanglement?^12^,^13^,^14^. Negative answer for this question prevents the use of an entanglement based-on quantum protocol that utilizes such a channel. If entanglement does not vanish, one can ask for an exact quantification of the entanglement level of the final output state (see for example Mintert *et al.*^15^), where the answer indicates on the success rate of the quantum protocol.

In this work, we concentrate on the case when a maximally entangled two-qubit state experiences unital noise (i.e., the channel maps the maximally mixed state onto itself). For this case, the answers for the two above-mentioned questions might be different if the channel is applied on both qubits of the entangled pair (a *bilocal* unital channel), or if it is a *unilocal* unital channel that operates only on one qubit of the pair. The entanglement breaking condition for the unilocal case was first derived by M. B. Ruskai^12^, where the calculation of the output state entanglement level is presented in the work of Ziman *et al.*^13^. Assuming an uncorrelated application of the same unital channel bilocally, the answer for the first question, is given in the work of Filippov *et al.*^14^. Here, we address the complementary answer for the second question when the noise is bilocal. We quantify the entanglement level for the specific bilocal unital Pauli channel and an initial maximally entangled Bell state, which is in the same basis. We show that the entanglement level of the bipartite system, when quantified by the concurrence measure, is described by simple relations of the important parameters of the noise. Both unilocal and bilocal dynamics of maximally entangled states are verified experimentally using an all-optical setup. We have also measured, the unilocal dynamics of states that are partially entangled, and show how entanglement evolution is affected by the amount of entanglement in the initial state, and its mixedness.

## Theoretical model

The entanglement of a qubit pair is commonly quantified using the concurrence measure^16^ defined as 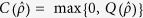, where 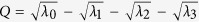, *λ*_*i*_ are the ordered positive eigenvalues of 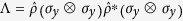, 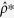 is the complex conjugate of the density matrix 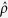 of the state in the computational basis and *σ*_*y*_ is the second Pauli matrix. The concurrence is one for maximally entangled states such as the Bell states 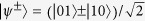 and 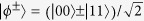, and zero for separable states.

Consider a quantum channel that acts on a single-qubit state 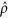. The operation of the channel can be uniquely described by a completely positive map 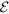, using the elements of the process matrix *χ*


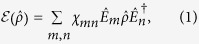


where 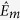 are called Kraus operators and span the vector space of 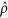. *χ* is positive, Hermitian, and satisfies *Tr*(*χ*) = 1 (i.e., the channel is nondissipative). In the case of a quantum system in *n*-dimensional Hilbert space, *χ* is a *n*^2^ × *n*^2^ matrix. In addition, the channel can be geometrically represented as the mapping of the surface of the Bloch sphere onto a smaller contained ellipsoid surface^1^.

If the channel is unital (i.e., 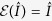), the sphere surface and the mapped ellipsoid are concentric. As was shown in Ref. 17, the mapping operation of unital channels can be understood as the implementation of two unitary rotations {*U*,*V*}, along with a three-parameter simpler map 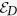:


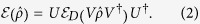


The map 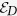 can be described using three parameters {*R*_1_,*R*_2_,*R*_3_}, which are the lengths of the primary axes of the mapped ellipsoid. For unital channels, the process matrix that describes the 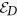 operation is *χ*_*D*_, the diagonalization of the process matrix *χ*. The radii {*R*_1_,*R*_2_,*R*_3_} are related to the eigenvalues of *χ* by


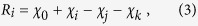


where *i* ≠ *j* ≠ *k* ≠ 0^17^. A negative value of *R*_*i*_ is interpreted as an inversion of the mapped ellipsoid with respect to a plane perpendicular to *R*_*i*_. The complete positivity of *χ* is equivalent to the requirements that |*R*_*i*_ ± *R*_*j*_| ≤ |1 ± *R*_*k*_|^17^. These inequalities define an allowed tetrahedral volume within the three dimensional radii space.

A unital channel that does not include rotations (i.e., *U* = *V* = *I*) is called a Pauli channel. When the 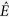 matrices are the *σ*_0_ identity matrix and the *σ*_1_, *σ*_2_, and *σ*_3_ Pauli matrices, the *χ* matrix that describes the Pauli channel is the diagonal *χ*_*D*_ matrix. Denoting the corresponding eigenvalues of *χ*_*D*_ by {*χ*_0_, .., *χ*_3_}, we write the probability for a change in the initial state as


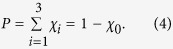


We analyze the case when a qubit pair is initially prepared in a maximally entangled Bell state |*ψ*_*B*_〉. A unital Pauli channel, which is designated by the symbol $ and represented by a diagonal matrix *χ*_*D*_, is then applied with a probability *P* to one of the two qubits (i.e., 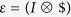). Calculating the output state 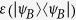 using Eq. (1), we obtain that the eigenvalues of the corresponding Λ[(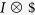)|*ψ*_*B*_〉〈*ψ*_*B*_|] matrix are 
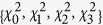
. If 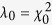 is the maximal eigenvalue, the concurrence in terms of *P* is written using Eq. (4) as: *C* = max{*χ*_0_ − *χ*_1_ − *χ*_2_ − *χ*_3_,0} = max{1 − 2*P*,0}.

Rewriting the concurrence as a function of the primary radii *R*_*i*_, we obtain: *C* = max{(*R*_1_ + *R*_2_ + *R*_3_ − 1)/2,0}. Generalizing this relation to the case where the maximal eigenvalue of Λ is not *λ*_0_, the concurrence becomes


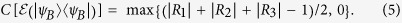


This equation describes the concurrence evolution when the noise is unital, and the initial state is a Bell state^13^. As unitary local rotations do not change the amount of entanglement, Eq. (5) is valid not just for the Bell states, but also for any maximally entangled initial state that experiences a unilocal unital noise. Regarding the question of the entanglement preservation, an immediate result from Eq. (5) is that the entanglement of any initial maximally entangled state vanishes when |*R*_1_| + |*R*_2_| + |*R*_3_| ≤ 1. Based on the presented calculation, we can not conclude that when |*R*_1_| + |*R*_2_| + |*R*_3_| ≤ 1, the channel is an entanglement breaking channel (i.e., the systems become separable for any initial state)^18^. Nevertheless, this condition coincides with the entanglement breaking condition for the general unilocal case^12^.

A second situation that we would like to study is when the same local unital Pauli process $ is applied to both qubits of |*ψ*_*B*_〉 (i.e., 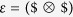). Calculating the concurrence of the output state as a function of the eigenvalues of *χ* {*χ*_0_, .., *χ*_3_}, we find that the eigenvalues of Λ[(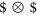)|*ψ*_*B*_〉〈*ψ*_*B*_|] are


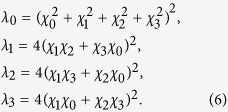


*λ*_0_ is the maximal eigenvalue of Λ. Substituting the values of *R*_*i*_ from Eq. (3) results with the output state concurrence


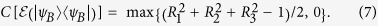


Notice the similarity between our result of Eq. (7) and the former result of Eq. (5). In a similar way, we can deduce that when 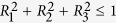, entanglement is annihilated if the initial state is a Bell state. This is in accordance with the entanglement annihilating condition that was reported in Ref. 14 as applicable to the bilocal case of unital channels that are applied on any entangled state. Unlike Eq. (5), the entanglement dynamics described by Eq. (7) does not apply to *any* unital channel and to *any* maximally entangled initial state. Numerical simulations suggest that in the more general case where the bilocal unital Pauli channel is applied on a maximally entangled state which is not a Bell state, the degradation of the concurrence is faster, and 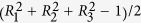 is only an upper bound for the concurrence. We note that Eq. (7) does hold in some important cases, such as when the noise is isotropic (*R*_1_ = *R*_2_ = *R*_3_, as will be shown below) and the initial state is any maximally entangled state, or for any unital noise when the initial state is the singlet state |*ψ*^−^〉.

## Experimental results

### Experimental setup

**Figure 1:**
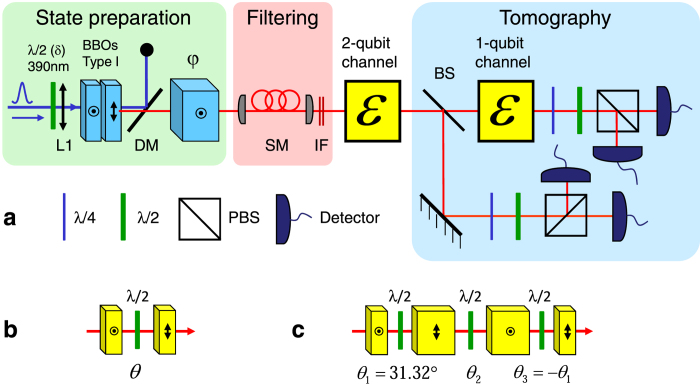
The experimental setup. (**a**) State generation and characterization units: photon pairs are generated in the BBO crystals, which are located after a lens (L1) and a half-wave plate (HWP, *λ*/2) in an angle of *δ*. The down-converted signal passes through a dichroic mirror (DM), a birefringent compensating crystal (*φ*), a single-mode fiber (SM), and a 3 nm interference bandpass filter (IF). In the state characterization unit, the photons are split probabilistically by a beam splitter (BS) to two ports. In each port, the photons pass a sequence of a quarter-wave plate (*λ*/4), a HWP, and a polarizing beam splitter (PBS) before being coupled into single-photon detectors. (**b**) The two-field channel, composed of two perpendicularly oriented identical 2 mm thick calcite crystals. (**c**) The three-field (isotropic) channel. This channel is composed of four crystals and two fixed HWPs. The thickness of the two outer (inner) crystals is 1 mm (2 mm).

In order to study entanglement dynamics experimentally, we generated entangled pairs of photons, transmitted them through controllable unital noisy channels, and measured the final concurrence of the output states. Entanglement was formed between the polarization degrees of freedom (DOFs), where the horizontal |*h*〉 and the vertical |*v*〉 polarization modes define the computational basis. The experimental setup is shown in Fig. 1a (A full description appears in the method section). A pulsed laser pumps nonlinear crystals, and generates collinear photon pairs in the process of spontaneous parametric down conversion. The generated photons are in the state |*ψ*〉 = cos(2*δ*)|*hh*〉 + sin(2*δ*)*e*^*i^φ^*^|*vv*〉, where the angle *δ* is the angle of a half-wave plate (HWP) that controls the pump beam polarization. Tilting a birefringent crystal that is placed after the generating crystals for temporal compensation reasons, controls the angle *φ*. In the characterization unit, the photon-pair is probabilistically split using a beam splitter (BS). Then, the post-selected two-port polarization state is measured by a two-qubit quantum state tomography procedure^19^.

Controlled quantum channels were implemented using a sequence of fixed birefringent calcite crystals and HWPs^20^,^21^ (see Fig. 1b,c). Each crystal entangles the polarization modes of a photon with its internal temporal DOFs. Decoherence occurs when the photon detection is insensitive to the temporal delays, practically averaging over these DOFs. In order to apply a channel unilocally we placed it in one port after the BS. A bilocal channel was realized by placing the channel before the BS. For both channel types, control over the noise probability *P* was achieved by rotating the corresponding HWPs to different angle settings^21^.

Two different unital channels were implemented. The first is the two-field channel^21^,^22^, (see Fig. 1b). It is described by random, but equally probable *σ*_1_ or *σ*_2_ rotations of the initial state, with overall noise probability of *P*


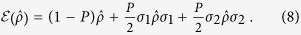


Substituting the process matrix eigenvalues 
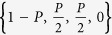
 in Eq. (3), we find that the primary radii of the mapped ellipsoid follows {*R*_1_ = *R*_2_, *R*_3_ = 2*R*_1_ − 1}. The second channel is an isotropic depolarization channel^23^ (see Fig. 1c). It is described as


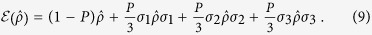


### Dynamics of maximally entangled states

**Figure 2:**
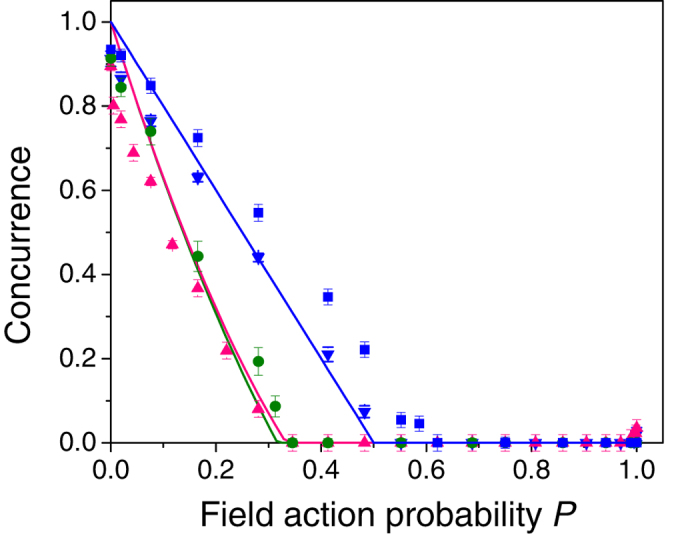
Measured entanglement dynamics of a |*ϕ*+〉 state. Four cases are presented: The two-field channel, when applied to one photon (downward blue triangles) or to both (upward pink triangles), and the isotropic channel, when applied to one photon (blue squares) or to both (green circles). Solid lines represent the theoretical predictions.

**Figure 3:**
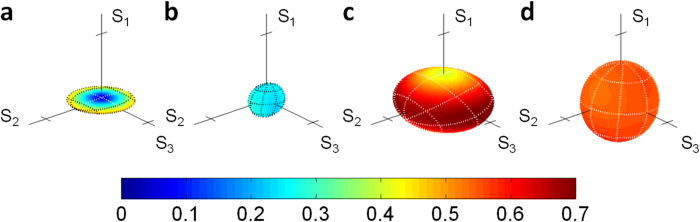
Reconstruction of the measured entanglement breaking processes in the Bloch sphere representation. (**a**) and (**b**) correspond to the two-field and the isotropic channels when applied to only one of the photons, respectively. (**c**) and (**d**) represent the cases when the two-field and the isotropic channels are applied to both photons, respectively. Ellipsoid surface colors show the distance from the origin. Axis ticks are at values of 1.

Setting *δ* = 22.5° and *φ* = 0, we generated |*ϕ*^+^ 〉 Bell states with an initial concurrence of 0.90 ± 0.01. Either one or both photons were transmitted through the two types of channels, as described before. For each decoherence setting, the output state concurrence is calculated from the reconstructed density matrix. It is presented in Fig. 2 as a function of the noise probability *P*, along with the theoretical predictions. When either the two-field or the isotropic channels are applied to one of the two qubits, the concurrence is degrading similarly as a linear function of *P*. Entanglement breaking should occur when 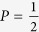. Reconstructed processes of the measured entanglement breaking points are presented in Fig. 3 using the Bloch sphere representation: A two-field process of *P* = 0.52 ± 0.01 is shown in Fig. 3a, and an isotropic process of *P* = 0.59 ± 0.01 is shown in Fig. 3b. According to theoretical calculations, if the channels are applied to both qubits, the concurrence dynamics is quadratic with *P*. Here, for the two processes, the dynamics is close but not identical. Entanglement annihilation for the two-field channel occurs when 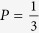, and for the isotropic channel when 
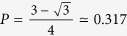
, which corresponds to a mapped sphere with radius of 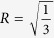. The corresponding measured processes for the two-field channel (*P* = 0.31 ± 0.01), and for the isotropic channel (*P* = 0.35 ± 0.01) are presented in Fig. 3c,d, respectively.

As predicted, when the channels are applied bilocally, concurrence vanishes faster than when applied unilocally. The deviation from theory is larger for the isotropic channel, where decoherence is slower than expected. We explain this as a result of insufficient temporal separation by the birefringent crystals^20^,^21^,^23^; In experiments, we used a signal with a coherence time of 300 fs. It is determined by the 3 nm wide bandpass filters that were used for spectral filtering. The two-field channel is using two 2 mm wide calcite crystals, thus the temporal separation between every sequential temporal modes was ~1,140 fs. On the other hand, when realizing the four-crystal configuration for the isotropic channel, we have also used two 1 mm crystals since the configuration requires a different width for two of the crystals. Thus, the minimal temporal delay between two sequential modes was only ~570 fs - not enough to prevent any overlap between the two modes. This overlap reduced and delayed the decoherence effects of the channel. It can be seen from Fig. 2 that the deviation from theory in the isotropic case is more significant when the channel is applied on one photon. This is a result of the difference between the linear dependency and the quadratic dependency of the concurrence in *P*, when the noise is applied to one or two photons, respectively. Even though the dynamics deviates from theory, it is still linear or quadratic, according to Eqs. (5) and (7). Thus, for the same concurrence value, the deviation in the quadratic case should be smaller because the corresponding probability *P* values are less than 1, and they are also smaller than the corresponding *P* values for the linear case.

### Dynamics of initial partially entangled states

In addition to studying the dynamics of initial maximally entangled states, we investigated the evolution of partially entangled states (PES). We now focus on the case of an isotropic channel which is applied only to one of the qubits. Two different classes of initial states were considered: pure PES and mixed PES. Different pure PES are generated by adjusting the HWP angle *δ*, so that the crystals are not equally pumped. The initial state concurrence is *C* = sin(4*δ*). Specifically, we applied isotropic noise to states with initial concurrence of 0.50 ± 0.01 and 0.16 ± 0.01. When applied to pure PES, the concurrence dynamics should evolve according to the factorization law, derived by Konrad *et al.*^7^:


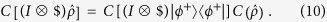


This relation states that the concurrence of any initial pure state after the application of a channel $ on one of the qubits can be factorized into the initial state concurrence and the concurrence that results when the same channel is applied to a pure Bell state.

**Figure 4:**
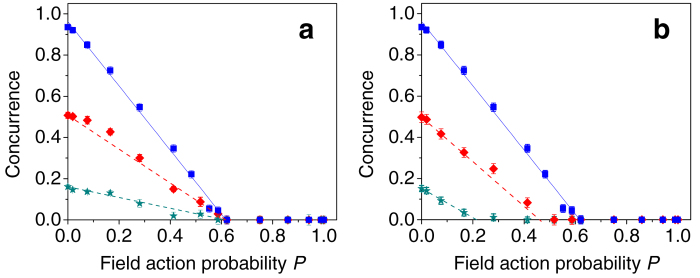
PES dynamics. Experimental results of the entanglement dynamics when the isotropic channel is applied to one qubit of initially (**a**) pure and (**b**) mixed PES.

The results for the initially pure PES are presented in Fig. 4a, along with the corresponding dynamics of an initial |*ϕ*^+^ 〉 state that also appears in Fig. 2. The solid line in Fig. 4a represents a linear fit for the measured dynamics of the |*ϕ*^+^ 〉 state. As was stated before, theory predicts a linear dependency, but entanglement breaking at *P* = 0.5. Because of experimental errors, the concurrence fit reaches zero only at *P* = 0.62 ± 0.02. From Eq. (10) it is clear that also the concurrence of the pure PES should have a linear dependency on *P*. We draw the straight dashed lines that connect the initial state concurrence at *P* = 0 and the experimental entanglement breaking point. As can be seen in Fig. 4a, the dynamics of the two PES indeed follows the corresponding linear predictions, reaching zero concurrence at the same point.

We also studied initial PES that are partially mixed. The entanglement evolution of such states was derived as an extension to Eq. (10) by Jiménez-Farías *et al.*^8^. The initial mixed PES 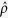 is expressed in terms of an additional channel $′ that is applied on one qubit of a pure two-qubit state 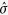 which is not necessarily maximally entangled: 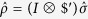 24. The concurrence of 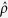 after a $ channel is applied to one of its qubits is


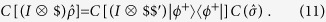


Mixed PES were generated by introducing partial dephasing to an initial |*ϕ*^+^ 〉 state as follows: the compensating birefringent crystal was replaced with shorter crystals that did not correct sufficiently the temporal walk-off between the horizontal and the vertical amplitudes of the initial state, effectively generating a dephasing noise^9^,^20^. in our case, where $′ is a dephasing channel, and $ is an isotropic channel, the concurrence also has a linear dependency on the isotropic noise probability *P*. Compared to the former case of initial pure states, decoherence occurs faster. As the initial state is more dephased, it will lose its entanglement earlier.

The experimentally measured dynamics of two initially mixed PES with initial concurrence of 0.50 ± 0.03 and 0.15 ± 0.01 is presented in Fig. 4b, together with the |*ϕ*^+^ 〉 previous results. The initial concurrence values are similar to those that were studied in the pure PES case. The solid straight line is the same fit to the |*ϕ*^+^ 〉 case as with the pure PES case. The theoretical lines for the other two cases were calculated numerically for the concurrence values of the initially mixed PES. The presented dashed lines are corrected according to the experimental deviation of the |*ϕ*^+^ 〉 case, i.e., their *P* values are multiplied by the ratio 0.62/0.5 between the measured and predicted *P* values for entanglement breaking.

The presented results demonstrate that the entanglement contained in mixed PES is more fragile to noise compared to that of pure PES with the same level of concurrence. As in the case of maximally entangled states (Fig. 2), most of the experimental deviation from theory is explained by the length of the shortest crystals of the isotropic channel. Additional deviation in the mixed PES case results from the overlap between the temporal modes of the initial dephasing channel and those created by the isotropic channels. Nevertheless, our results demonstrate the differences between the various cases very clearly.

## Discussion

In this work, we studied the dynamics of entangled states when subjected to unital noisy channels. We showed that concurrence, as an entanglement measure, is linked in a simple way to the principal radii of the ellipsoid that represents the noise map in the Bloch sphere picture. Explicitly, when the channel is applied on one of the qubits of a maximal entangled state (the unilocal case), the concurrence is linear with the sum of the absolute values of the ellipsoid radii. We derived the concurrence for the bilocal case when the same Pauli channel is applied to both qubits of a Bell state, and found that it has a similar dependence, but with the sum of the squares of the ellipsoid radii.

We realized two different channels using birefringent crystals. These channels were applied to either one or to both photons of a polarization entangled photon pair, in order to experimentally demonstrate the entanglement dynamics of maximally entangled polarization states. In the case of a unilocal isotropic noise, we also compared the entanglement degradation of initially pure and mixed partially entangled states. For states of similar initial concurrence, we have shown that dynamics depends on the initial degree of mixedness.

Two interesting issues that we leave open are the generalization of these results to non-unital channels and for systems of higher dimensionality. One may speculate: suppose a known unital channel operates on a three-qubit state that is maximally genuinely entangled. Can we quantify the entanglement of the output state using an entanglement measure which is proportional to the sum of the cubes of the ellipsoid radii that describe this channel?

## Methods

### Entangled states generation and characterization

Photon pairs are collinearly generated by the process of spontaneous parametric down conversion. Using a lens of 30 cm focal length (L1), a pulsed 390 nm pump laser is focused onto two perpendicularly oriented 1 mm thick type-I *β* — BaB_2_O_4_ (BBO) crystals. After the crystals, the down-converted signal is separated from the pump beam using a dichroic mirror (DM). A half-wave plate at an angle of *δ* is placed before the generating crystals in order to control the relative pump power of each crystal. Thus, the generated state is |*ψ*〉 = cos(2*δ*)|*hh*〉 + sin(2*δ*)*e*^*iφ*^|*vv*〉, where another compensating crystal which is placed after the generating crystals can control the *φ* angle. Before entering the quantum channel, the state is filtered spatially using a single-mode fiber (SM), and spectrally by a 3 nm interference bandpass filter (IF). In the state characterization unit, the photons are split probabilistically by a beam splitter (BS). The required projections for the quantum state tomography procedure are achieved using wave-plates and polarizing beam splitters (PBS) that are placed before the single-photon detectors of each port.

### Characterization of the channels

**Figure 5:**
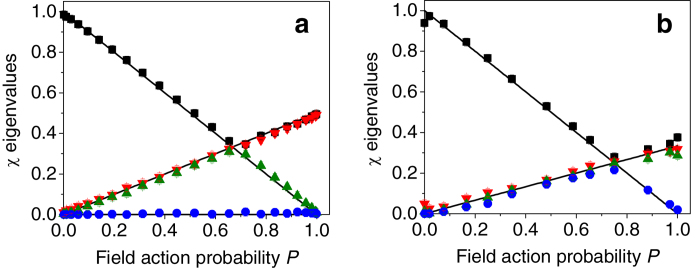
Channel characterization. Measured eigenvalues of the process matrices for (**a**) the two-field and (**b**) the isotropic channels as a function of the noise probability *P*, with their theoretical predictions (solid lines).

The dynamics of the eigenvalues of the single-qubit process matrix of the implemented channels, as obtained with a quantum process tomography procedure^1^ are presented in Fig. 5a,b. The noise probability *P* is controlled by the rotation of the corresponding channel wave-plate. Surprisingly, for both channels *P* = sin^2^(2*θ*), where for the isotropic channel *θ* = *θ*_2_. Errors are calculated using a maximal likelihood procedure and Monte Carlo simulations, assuming Poissonian noise^19^,^22^. Observing Fig. 5, it can be seen that the single-qubit channels where implemented with a high fidelity to the theory. For the two-field channel, typical fidelity values were 97 ± 2%, while for for the isotropic channel, the fidelity between the implemented and the ideal channels was 98 ± 2%. We note that these measurements were conducted using a 5 nm interference bandpass filter, while the measurements of two-photon states described in the main text were performed using a 3 nm interference bandpass filter. Consequently, the coherence time of the photons that were used to characterize the channels was shorter than that of the two-photon states. Thus, the operation of the channels on the two-photon states (and especially that of the isotropic channel, see main text) was with lower fidelity to an ideal process. Nevertheless, the channels still induced unital noise since they always operate symmetrically on orthogonal polarizations.

## Additional Information

**How to cite this article**: Shaham, A. *et al.* Entanglement dynamics in the presence of controlled unital noise. *Sci. Rep.* **5**, 10796; doi: 10.1038/srep10796 (2015).
